# ‘If not TB, what could it be?’ Chest X-ray findings from the 2016 Kenya Tuberculosis Prevalence Survey

**DOI:** 10.1136/thoraxjnl-2020-216123

**Published:** 2021-01-27

**Authors:** Brenda Nyambura Mungai, Elizabeth Joekes, Enos Masini, Angela Obasi, Veronica Manduku, Beatrice Mugi, Jane Ong’angò, Dickson Kirathe, Richard Kiplimo, Joseph Sitienei, Rose Oronje, Ben Morton, Stephen Bertel Squire, Peter MacPherson, IMPALA Consortium, Emmanuel Addo-Yobo

**Affiliations:** 1 Department of Clinical Sciences, Liverpool School of Tropical Medicine, Liverpool, UK; 2 Worldwide Radiology, Liverpool, UK; 3 The Global Fund to Fight AIDS, Tuberculosis and Malaria, Geneva, Switzerland; 4 Stop TB Partnership, Geneva, Switzerland; 5 Department of International Public Health, Liverpool School of Tropical Medicine, Liverpool, UK; 6 Axess Sexual Health, Liverpool University Hospitals NHS Foundation Trust, Liverpool, UK; 7 Kenya Medical Research Institute, Nairobi, Kenya; 8 Kenyatta National Hospital, Nairobi, Kenya; 9 Division of National Tuberculosis, Leprosy and Lung Disease Program, Nairobi, Kenya; 10 African Institute for Development Policy, Nairobi, Kenya; 11 Malawi-Liverpool-Wellcome Trust Clinical Research Programme, Blantyre, Malawi; 12 Critical Care, Liverpool University Hospitals NHS Foundation Trust, Liverpool, Liverpool, UK; 13 Tropical & Infectious Diseases Unit, Liverpool University Hospitals NHS Foundation Trust, Liverpool, UK; 14 Department of Clinical Research, London School of Hygiene and Tropical Medicine, London, UK

**Keywords:** tuberculosis, imaging/CT MRI etc, bronchiectasis, COPD epidemiology, emphysema

## Abstract

**Background:**

The prevalence of diseases other than TB detected during chest X-ray (CXR) screening is unknown in sub-Saharan Africa. This represents a missed opportunity for identification and treatment of potentially significant disease. Our aim was to describe and quantify non-TB abnormalities identified by TB-focused CXR screening during the 2016 Kenya National TB Prevalence Survey.

**Methods:**

We reviewed a random sample of 1140 adult (≥15 years) CXRs classified as ‘abnormal, suggestive of TB’ or ‘abnormal other’ during field interpretation from the TB prevalence survey. Each image was read (blinded to field classification and study radiologist read) by two expert radiologists, with images classified into one of four major anatomical categories and primary radiological findings. A third reader resolved discrepancies. Prevalence and 95% CIs of abnormalities diagnosis were estimated.

**Findings:**

Cardiomegaly was the most common non-TB abnormality at 259 out of 1123 (23.1%, 95% CI 20.6% to 25.6%), while cardiomegaly with features of cardiac failure occurred in 17 out of 1123 (1.5%, 95% CI 0.9% to 2.4%). We also identified chronic pulmonary pathology including suspected COPD in 3.2% (95% CI 2.3% to 4.4%) and non-specific patterns in 4.6% (95% CI 3.5% to 6.0%). Prevalence of active-TB and severe post-TB lung changes was 3.6% (95% CI 2.6% to 4.8%) and 1.4% (95% CI 0.8% to 2.3%), respectively.

**Interpretation:**

Based on radiological findings, we identified a wide variety of non-TB abnormalities during population-based TB screening. TB prevalence surveys and active case finding activities using mass CXR offer an opportunity to integrate disease screening efforts.

**Funding:**

National Institute for Health Research (IMPALA-grant reference 16/136/35).

**Key messages**

**What is the key question?**

- What was the prevalence of non-TB findings when chest X-rays (CXRs) were used in the 2016 Kenya National TB Prevalence Survey?

**What is the bottom line?**

- There was a high prevalence of cardiomegaly, chronic pulmonary diseases, post-TB lung disease and non-specific lung diseases; implementation of CXR TB screening in lower-income and middle-income countries offers an opportunity to integrate disease screening efforts.

**Why read on?**

- To our knowledge, this is the first study in sub-Saharan Africa to describe and quantify non-TB CXR findings among participants who underwent mass screening as part of a population-based TB prevalence survey and has important health policy implications.

## Introduction

TB remains the leading adult infectious killer in the world.^1^ Despite an estimated nine per cent relative increase in case detection in 2018, there are still three million (30%) people with TB who are undiagnosed or not reported to national TB programmes.^1^ In an effort to identify missing people with TB, countries have adopted more sensitive diagnostic tools, including Xpert MTB/RIF, scaled up intensified active case finding (ACF), and adapted their screening and diagnostic algorithms to include chest X-ray (CXR) as a sensitive and efficient high-throughput initial screening test.^2^

Historically, miniature radiography for mass TB screening activities was widely used in high-income countries throughout the 20th century.^3–6^ In lower-income and middle-income countries (LMICs), however, CXR has been used primarily as a complementary tool to support clinical diagnosis of patients who are sputum smear negative.^7^ Following the findings from national TB prevalence surveys that have employed CXR for screening, there is a renewed interest in the utility of CXR for TB screening, and to triage people seeking care with symptoms for further TB investigations.^8–10^ In TB prevalence surveys conducted in LMICs, CXR has shown high sensitivity for pulmonary TB (PTB) (94%, 95% CI 88% to 98%) but poor specificity (73%, 95% CI 68% to 77%), necessitating confirmation with a microbiological test.^11–13^ Mathematical modelling of various TB screening algorithms, as well as diagnostic algorithms, shows that CXR followed by Xpert MTB/RIF, though resource intensive, has the lowest number needed to screen to identify a case.^14^ Increased digital CXR availability, coupled with the development of computer aided detection (CAD) software for identification of TB, has enabled increasing use of CXR in screening for TB in areas with limited access to radiologists or expert clinicians.^15 16^

Use of CXR for mass TB screening will identify other conditions, especially those related to complications of a rising burden of non-communicable diseases (NCDs) in LMICs, including cardiovascular disease, chronic respiratory disease and cancer.^17^ A short narrative report from Europe in the 1940s highlighted a significant number of 1225 out of 3423 (35.7%) of non-TB findings in mass radiography screening.^5^ However, there is no contemporaneous evidence about the prevalence of non-TB abnormalities identified during TB prevalence surveys and mass radiographic TB screening interventions. TB prevalence surveys focus on accurate estimation of TB prevalence encouraging readers to prioritise sensitivity over specificity and include any CXR abnormalities to identify participants eligible for TB bacteriological testing.^9^

Systematic screening/ACF programmes also focus on early detection of active TB.^4^ CAD software for TB) demonstrates high sensitivity for CXR TB diagnosis but is not calibrated for detection of non-TB abnormalities.^18^ Countries are currently adopting CXR screening in combination with CAD software systems, both for mass community TB screening activities and in healthcare settings.^16^ However, the burden of non-TB CXR abnormalities during these mass screening activities is unknown.

We set out to describe and quantify the nature of non-TB abnormalities on abnormal CXRs taken during the 2016 Kenya National TB Prevalence Survey.^13^ We hypothesised that the use of CXR during TB screening would identify a substantial number of people with non-TB abnormalities who may require further clinical attention.

## Materials and methods

### Study design

We conducted a secondary retrospective analysis on cross-sectional study data^13^ using individual-level participant CXR data from adult community members who took part in the 2016 Kenya National TB Prevalence Survey.^13^ The study was in two parts: an initial pilot study (n=484) to refine tools and estimate the full sample size required for precision and the main study (n=1140). The main aim was to estimate prevalence and uncertainty for CXR-identified non-TB disease pathology within this population.

### Study population

The prevalence survey (reported elsewhere)^12 13^ was a population-based cross-sectional study conducted in 2015–2016. The aim was to determine the prevalence of bacteriologically confirmed PTB among adults (≥15 years) and to assess health-seeking behaviour. The survey used the WHO recommended screening strategy comprising symptom questionnaire and CXR.^9^ There were 63 050 enrolled participants, 62 484 (99%) underwent CXR screening. The survey identified 305 TB cases; weighted national prevalence of 558 (95% CI 455 to 662) per 100 000 adult population.^12 13^

### Methods

#### Classification of CXRs during the Kenya TB Prevalence Survey

Digitally acquired posterior-anterior CXRs were uploaded to a digital archive. Independent, blinded reading of each film was conducted by two clinical officers in the field who had undergone CXR training. Each image was classified as either (1) normal; (2) ‘abnormal, suggestive of TB’; or (3) ‘abnormal other’. Any participant with a CXR classified as ‘abnormal, suggestive of TB’ by either one of the clinical officers, or with a cough of more than 2 weeks, was eligible for sputum collection. Those confirmed to have TB were referred for treatment and those with other CXR abnormalities were to be linked to a health facility. The prevalence survey has been reported fully elsewhere.^13^

##### Study procedures

We obtained an anonymised line list of all the participants from the prevalence survey database; our sampling frame included all CXRs classified as ‘abnormal, suggestive of TB’ or ‘abnormal other’ by the survey field readers. Images selected for inclusion in this study were uploaded to a web-based picture archiving and communication system.

Ten specialist radiologists (five Kenyan, five from the UK) with median experience of 12.5 years in TB/chest radiology were recruited (online supplemental appendix 1). One-to-one training of the radiologists was provided on the online reporting tool and diagnostic case definitions.^19^ Based on previous reporting tools, we developed radiological definitions, comprised of abnormalities within four major anatomical areas (lung parenchyma, heart and great vessels, pleura and mediastinum).^20^ For each major anatomical area, a list of most common radiological diagnoses was developed, taking into consideration Kenyan disease epidemiology (online supplemental appendix 2). Radiologists were able to select one primary diagnosis. Differential diagnoses could be added where a single, confident primary diagnosis could not be made.

10.1136/thoraxjnl-2020-216123.supp1 Supplementary data

Each radiologist was randomly assigned CXRs for review. After completion of each reading, the image was released into a pool for second reading. The readers were blinded to each other’s report, but not to clinical information (sex, age, HIV status and symptoms). Finally, 10% of images were reallocated to the original readers for assessment of intraobserver variation. Where pairs of radiologists had discrepant primary diagnoses, one of two additional radiologists undertook a consensus read, with knowledge of the first two radiologists’ classification.

### Data management and analysis

Based on the pilot study findings (online supplemental appendix 3), 1140 CXR images (390 ‘abnormal, suggestive of TB’ and 750 ‘abnormal other’) would be required to estimate the prevalence of old/latent TB and cardiomegaly (the most common findings in the pilot study in the respective categories) within 3.5% percentage points of the true value with 95% confidence.

For the main study, we included images classified as either ‘abnormal, suggestive of TB’ and ‘abnormal other’, excluding those sampled in the pilot study. These images were grouped into 99 strata as per the prevalence survey clusters, and sampled without replacement from each stratum. Statistical analysis used R V.3.6.2 (The R Foundation for Statistical Computing, Vienna, Austria). Inter-reader and intrareader agreement was calculated using the Cohen’s Kappa statistic. Study participant characteristics were calculated as medians or percentages. The prevalence of primary diagnoses was calculated as the number of CXRs depicting the abnormality divided by the total number of images that were readable; 95% CIs were estimated using the binomial exact method. A number of final diagnoses were combined or removed before analysis, based on their prevalence and likelihood of clinical relevance.

## Results

**Figure 1:**
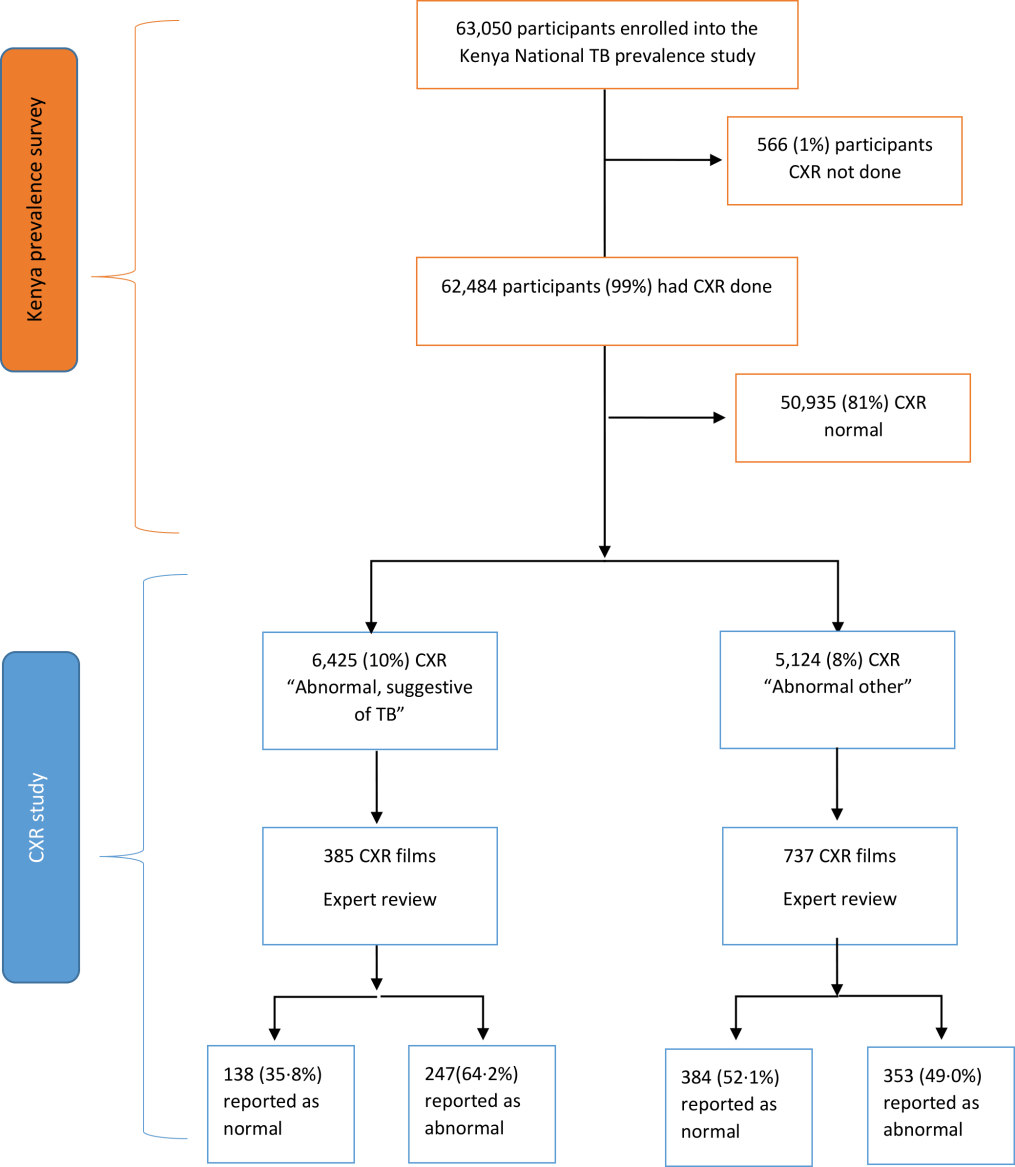
Consolidated Standards of Reporting Trials (CONSORT) flow diagram showing participant flow from the Kenya National TB Prevalence Survey and this nested chest X-ray (CXR) study.

Out of 63 050 participants in the prevalence survey, 62 484 (99%) underwent CXR, with 50 935 (81.5%) reported as normal by survey field staff, 6425 (10.3%) as ‘abnormal, suggestive of TB’ and 5124 (8.2%) as ‘abnormal other’ (figure 1).

### Participant characteristics

Out of 1140 images included in the main study, 1123 (98.5%) were read by two radiologists and 17 (1.5%) were classified as unreadable. The median patient age was 51.0 years (IQR 36.5–66.0) and 720 (64.2%) were female. Two hundred and fifty (22.3%) had reported cough of any duration, 40 (3.6%) chest pain and 42 (3.7%) self-reported HIV-positive status. Six (0.5%) reported current and 34 (3.0%) reported previous TB treatment. GeneXpert and/or culture results were positive for 27 out of 456 (5.9%) of those that had sputum tested (table 1).

**Table 1:** Characteristics and sputum microbiology results of study participants, stratified by the original survey classification of the chest X-rays as ‘abnormal, suggestive of TB’ versus ‘abnormal other’

	Abnormal, suggestive of TB (n=385)	Abnormal, other (n=737)	Total (n=1123)	P value
Sex				<0.001
Missing	0	1	1	
Female	198 (51.4%)	570 (70.8%)	720 (64.2%)	
Male	187 (48.6%)	239 (29.2%)	402 (35.8%)	
Age (years)				<0.001
Median (Q1, Q3)	45.0 (33.0, 64.0)	54.0 (38.0, 67.0)	51.0 (36.2, 66.0)	
Study consensus interpretation				<0.001
Abnormal CXR	247 (64.2%)	353 (49.0%)	600 (53.4%)	
Normal CXR	138 (35.8%)	384 (52.1%)	522 (46.6%)	
Missing			1	
Cough of any duration				0.002
Missing	0	1	1	
No	278 (72.2%)	594 (80.6%)	872 (77.7%)	
Yes	107 (27.8%)	143 (19.4%)	250 (22.3%)	
Reported weight loss				0.674
No	324 (84.2%)	613 (83.2%)	937 (83.5%)	
Yes	61 (15.8%)	124 (16.8%)	185 (16.5%)	
Reported fever				0.385
No	320 (83.1%)	597 (81.0%)	917 (81.7%)	
Yes	65 (16.9%)	140 (19.0%)	205 (18.3%)	
Reported night sweats				0.001
No	278 (72.2%)	594 (80.6%)	872 (77.7%)	
Yes	107 (27.8%)	143 (19.4%)	250 (22.3%)	
Self-reported HIV status				0.005
Missing	183	389	572	
HIV-negative	180 (89.1%)	328 (94.3%)	508 (92.4%)	
HIV-positive	22 (10.9%)	20 (5.7%)	42 (7.6%)	
Taking TB treatment				0.069
Missing	182	340	522	
No	199 (98.0%)	395 (99.5%)	594 (99.0%)	
Yes	4 (2.0%)	2 (0.5%)	6 (1.0%)	
Previously treated for TB				<0.001
Missing	182	403	522	
No	175 (86.2%)	391 (98.5%)	566 (94.3%)	
Yes	28 (13.8%)	6 (1.5%)	34 (5.7%)	
Sputum Xpert/culture result				0.001
Missing	66	600	666	
Negative	293 (91.8%)	185 (99.3%)	429 (94.1%)	
Positive	26 (8.2%)	1 (0.7%)	27 (5.9%)	

CXR, chest X-ray.

### Inter-reader and intrareader variability

The overall agreement between pairs of readers was moderate with kappa=0.41 (online supplemental appendix 4). There was perfect intrareader agreement at kappa=1. The median time between intraobserver reliability assessments was 22 days.

### Prevalence of CXR abnormalities

Overall, six hundred (53.4%) images were classified by study radiologists as having any abnormality. Of the images classified as ‘abnormal, suggestive of TB’ by field interpretation in the survey, 203 (64.9%) were classed by expert reviewers as abnormal, whereas among the ‘abnormal other’ category, 397 (49.0%) were abnormal by expert radiologist read (table 1).

**Figure 2:**
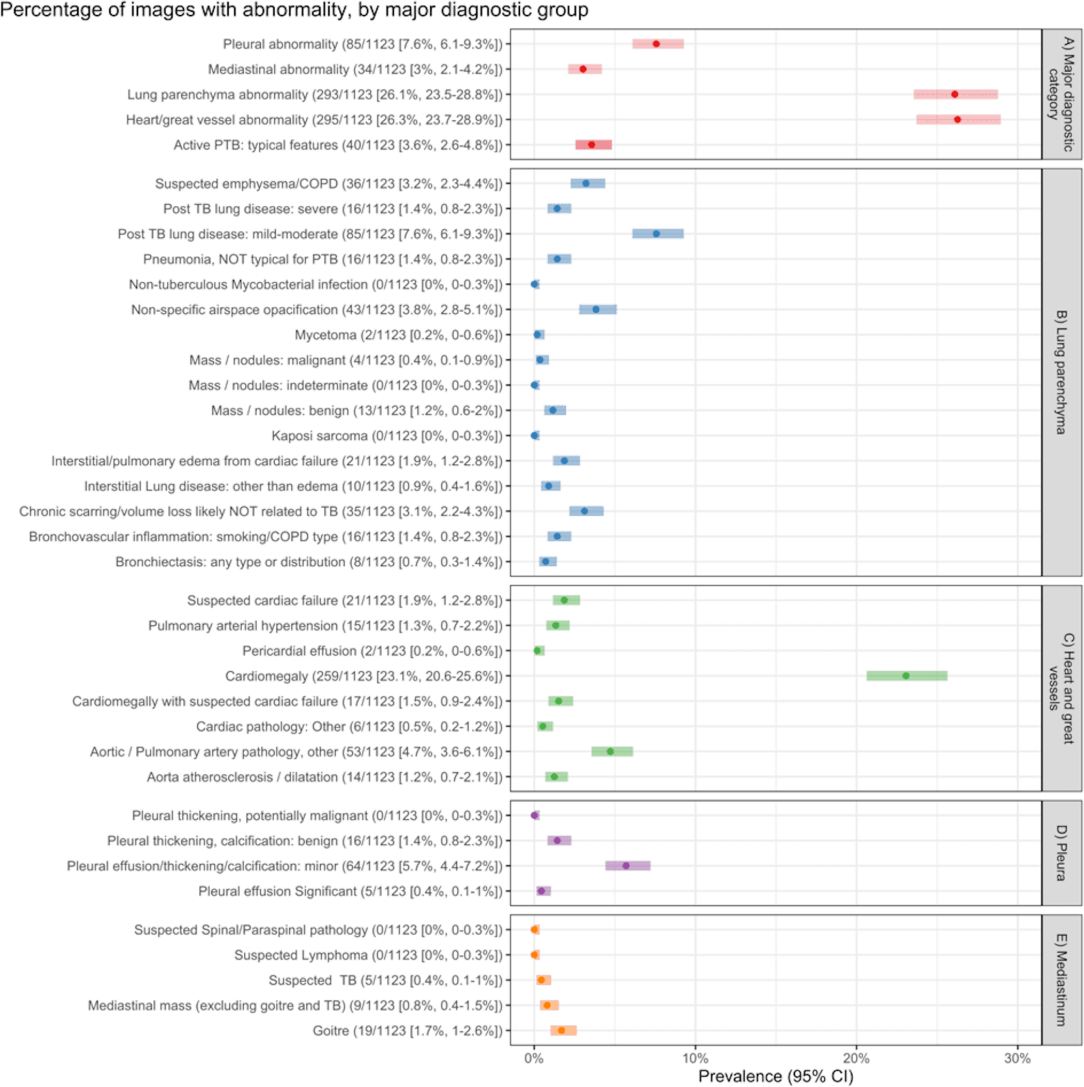
Prevalence of abnormalities by major diagnostic categories. PTB, pulmonary TB.

**Figure 3:**
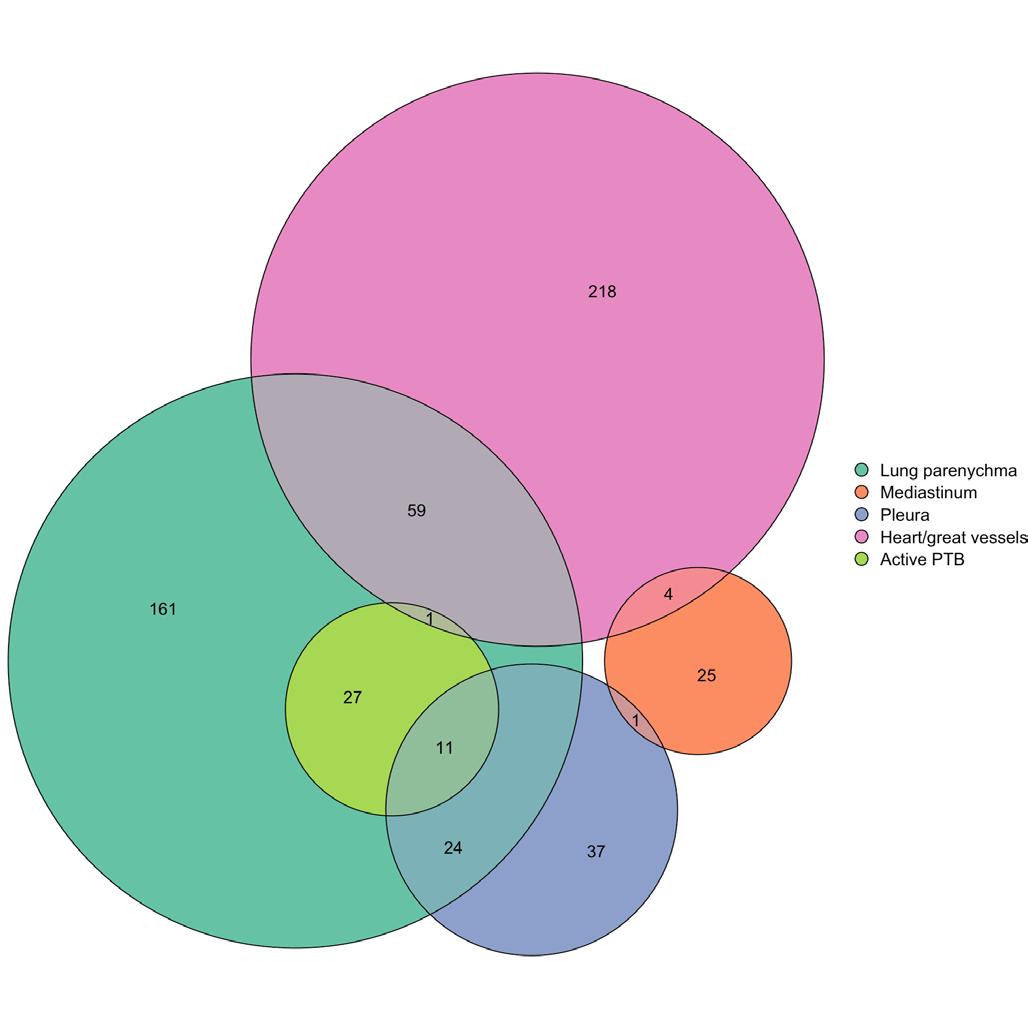
Euler diagram of abnormalities identified during the chest X-ray study.

Overall prevalence of abnormalities in the major anatomical categories were heart and/or great vessels 26.3% (95% CI 23.7% to 28.9%), lung parenchyma 26.1% (95% CI 23.5% to 28.8%), pleura 7.6% (95% CI 6.1% to 9.3%) and the mediastinum 3% (95% CI 2.1% to 4.2%) (figure 2). Among the 600 abnormal images, 21% (127/600) had multiple abnormalities, cardiomegaly accounted for 259 out of 600, 43.2% (39.2%–47.2%) followed by mild/moderate post-TB lung changes at 85 out of 600, 14.2% (11.5%–17.2%) (figure 3).

Among the potentially relevant non-TB abnormalities, cardiomegaly was the most prevalent at 23.1% (95% CI 20.6% to 25.6%), while cardiomegaly combined with features of cardiac failure occurred in 1.5% (95% CI 0.9% to 2.4%). Non-specific patterns were noted in 4.6% (95% CI 3.5% to 6.0%), while suspected COPD, including emphysema, was present in 3.2% (95% CI 2.3% to 4.4%). Mediastinal masses, excluding goitres, occurred in 0.8% (95% CI 0.4% to 1.5%).

For presumed TB-related abnormalities, prevalence of minor post-TB lung changes, such as old/latent TB involving fewer than two lobes of damage/scarring, was 7.6% (95% CI 6.1% to 9.3%), active TB was 3.6% (95% CI 2.6% to 4.8%) and severe post-TB lung changes, that is, bronchiectasis and/or destroyed lung, 1.4% (95% CI 0.8% to 2.3%).

Between a quarter and a third of females had cardiomegaly (29%). For males, 11.7% had cardiomegaly and 11.2% mild/moderate post-TB lung changes. History of cough was a common feature across all diagnosis types, with 69 out of 250 (27.6%) of coughers having cardiomegaly. Out of the 40 participants with chest pain, 22.5% (10.8%–38.5%) had cardiomegaly and 20% (9.1%–35.6%) had minor post-TB lung changes. Bacteriological confirmation of TB was found in all categories of reported TB-related lung abnormalities and 4 out 27 (14.8%, 4.2%–33.7%) of images reported as non-specific patterns (table 2). Out of the 34 participants with a history of previous TB treatment, features consistent with minor post-TB lung changes were reported in 11 (32.4%, 95% CI 17.4% to 50.5%), active PTB in 6 (17.6%, 95% CI 6.8% to 34.5%) and severe post-TB lung changes in 4 (11.8%, 95% CI 3.3% to 27.5%).

**Table 2:** Prevalence of potentially relevant radiological findings with accompanying characteristics

Radiological findings	Prevalence		Sex		Age	History of cough of any duration	History of chest pain	Bacteriological TB confirmation (Xpert/Culture)
	n=1123	%, 95% CI	Female n=721, % (95% CI)	Male n=402, % (95% CI)	Median, IQR	n=250, % (95% CI)	n=40, % (95% CI)	n=27, % (95% CI)
Cardiomegaly	259	23.1% (20.6% to 25.6%)	212/721, 29.4% (26.1% to 32.9%)	47/402, 11.7% (8.7% to 15.2%)	59 (45–70)	69/250, 27.6% (22.2% to 33.6%)	9/40, 22.5% (10.8% to 38.5%)	0/27, 0.0% (0.0% to 12.8%)
Cardiomegaly with heart failure	17	1.5% (0.9% to 2.4%)	12/721, 1.7% (0.9% to 2.9%)	5/402, 1.2% (0.4% to 2.9%)	77 (68–83)	7/250, 2.8% (1.1% to 5.7%)	0/40, 0.0% (0.0% to 8.8%)	0/27, 0.0% (0.0% to 12.8%)
Suspected cardiac failure	21	1.9% (1.2% to 2.8%)	16/721, 2.2% (1.3% to 3.6%)	5/402, 1.2% (0.4% to 2.9%)	75 (67–83)	8/250, 3.2% (1.4% to 6.2%)	0/40, 0.0% (0.0% to 8.8%)	0/27, 0.0% (0.0% to 12.8%)
Mild/moderate post-TB lung disease (*Old/latent TB with=<2 lobe damage*)	85	7.6% (6.1% to 9.3%)	40/721, 5.5% (4.0% to 7.5%)	45/402,11.2% (8.3% to 14.7%)	48 (37–66)	18/250, 7.2% (4.3% to 11.1%)	8/40, 20.0% (9.1% to 35.6%)	6/27, 22.2% (8.6% to 42.3%)
Non-specific opacification/interstitial pattern	52	4.6% (3.5% to 6.0%)	28/721, 3.9% (2.6% to 5.6%)	24/402, 6.0% (3.9% to 8.8%)	59 (46–76)	16/250, 6.4% (3.7% to 10.2%)	1/40, 2.5% (0.1% to 13.2%)	4/27, 14.8% (4.2% to 33.7%)
Active PTB	40	3.6% (2.6% to 4.8%)	15/721, 2.1% (1.2% to 3.4%)	25/402, 6.2% (4.1% to 9.0%)	36 (26–51)	21/250, 8.4% (5.3% to 12.6%)	4/40, 10.0% (2.8% to 23.7%)	11/27, 40.7% (22.4% to 61.2%)
Suspected emphysema/COPD	36	3.2% (2.3% to 4.4%)	16/721, 2.2% (1.3% to 3.6%)	20/402, 5.0% (3.1% to 7.6%)	68 (50–73)	11/250, 4.4% (2.2% to 7.7%)	0/40, 0.0% (0.0% to 8.8%)	0/27, 0.0% (0.0% to 12.8%)
Severe post-TB lung disease (*Bronchiectasis and or destroyed lung*)	16	1.4% (0.8% to 2.3%)	10/721, 1.4% (0.7% to 2.5%)	6/402, 1.5% (0.5% to 3.2%)	60 (34–72)	7/250, 2.8% (1.1% to 5.7%)	0/40, 0.0% (0.0% to 8.8%)	2/27, 7.4% (0.9% to 24.3%)
Mediastinal mass (excluding goitre/TB)	9	0.8% (0.4% to 1.5%)	7/721, 1.0% (0.4% to 2.0%)	0/402, 0.5% (0.1% to 1.8%)	61 (56–71)	2/250, 0.8% (0.1% to 2.9%)	0/40, 0.0% (0.0% to 8.8%)	0/27, 0.0% (0.0% to 12.8%)

## Discussion

The main finding from this analysis of X-ray images from the 2016 Kenya TB Prevalence Survey was that the use of CXR for TB population-based studies identified a large number of patients with non-TB-related abnormalities. Cardiac and pulmonary diseases accounted for 66% of the non-TB abnormalities in our setting. The radiological findings were consistent with complications of potential underlying NCDs. To our knowledge, this is the first study in sub-Saharan Africa to describe and quantify non-TB CXR findings among participants who underwent mass screening as part of a population-based TB prevalence survey. TB prevalence surveys and ACF activities using mass CXR offer an opportunity to identify other potential diseases; however, as currently structured, when TB is not confirmed, there is limited further diagnosis and management of these other conditions.^21^ The findings of our study are also timely in the wake of disruption in the health system caused by COVID-19.^22^ As countries accelerate ACF activities using CXR screening to make up for reductions in TB case-notification rates due to COVID-19, they could plan systems to manage other findings.

At the outset of the study, we expected abnormalities to be primarily related to non-TB pulmonary disease. However, the most prevalent findings were cardiac abnormalities with cardiomegaly at 23.1% (95% CI 20.6%–25.6%). An outpatient research clinic pilot study in The Gambia reports a similar finding of cardiac abnormalities being prevalent among patients without TB (n=108).^21^ The prevalence of cardiac abnormalities in our study is higher than in The Gambia (26.3% vs 20%).^21^ Cardiac abnormality diagnosis in The Gambia was through clinical assessment, ECG and echocardiography (as indicated) versus CXR only in our study. This, together with higher median age in our study compared with The Gambia (51 years vs 40 years), could explain our higher observed prevalence.

Calculation of the cardio-thoracic ratio (CTR) on CXR read by humans is a well-described affordable and reproducible screening method for cardiomegaly.^17 23^ However, current studies, including our own, use CTR cut-off values developed in Caucasian populations and there will be a need for robust validation of baseline CTR values for healthy populations in sub-Saharan Africa.^24^ CAD for detection of CTR is under development.^25^

In sub-Saharan Africa, the most common causes of cardiomegaly are conditions of significant public health importance associated with premature mortality, including hypertensive heart disease, cardiomyopathies, cor pulmonale, chronic rheumatic heart diseases and ischaemic heart diseases.^23 26^ Cardiomegaly has been associated with both higher body mass index and higher median systolic blood pressure.^17^ The high prevalence of cardiomegaly in our study supports exploration of the benefits of CVD screening during TB CXR screening as a potentially affordable public health intervention.^5 23^ Health messaging on prevention of NCDs through recommendations on diet, such as reduction of commercial sugar and high salt diet, could be considered for integration in such programmes.^23^

Non-TB-related respiratory pathology, including chronic respiratory diseases (CRD), was another significant finding in our cohort. In a Vancouver study in 1960s, three cases of significant previously unknown non-TB lung disease were identified for every new TB case; in our study, this figure was approximately 2:1.^6^ It should be noted that CXR alone has limited specificity for many of these conditions, especially in this cohort where very limited clinical information was available. The diagnoses of ‘non-specific airspace opacification’ and ‘interstitial pattern’ cover a range of possible pathologies, varying from incidental acute or chronic infective changes, not typical of TB, to non-infective pathology. COPD and emphysema cannot be diagnosed reliably on CXR alone, requiring spirometry and referral for further confirmation. However, CRD morbidity and mortality is on the rise, with the prevalence of COPD shown to range between 4% and 25% in one systematic review in sub-Saharan Africa comparable to 3.6% (2.3%–4.4%) in our study.^27^ As expected, our study confirmed that screening for TB will detect alternative lung abnormalities in a significant number of non-TB cases and spirometry as well as expert clinical review, for example, pulmonologists will be required for a subset of these patients.

Forty-four per cent of the participants in our study with reported post-TB lung changes had a history of TB treatment. Among those, CXR revealed bronchiectasis and/or destroyed lung in 11% (95% CI 3.3%–27.5%), which is lower than reported in a prospective study in Malawi, that used CT and reported >40% bronchiectasis and 10% lobar destruction post-TB treatment.^20^ Bronchiectasis has a lower detection rate on CXR than CT and will likely have been underestimated in our study. PTB is a risk factor for CRD and in the Malawi study ongoing clinical symptoms were associated with damage of three or more lobes,^20 28^ which is comparable to the ‘destroyed lung’ category in our CXR study. Unfortunately, patients with post TB lung disease (PTLD) and chronic symptoms are likely to be treated empirically for recurrent TB.^29^ We therefore recommend that TB screening programmes look into developing the area of PTLD further. Latent TB infection treatment should be offered to eligible patients with PTLD.^30^ Our study also identified other less common findings for which interventions may be costly. For example, mediastinal and lung masses/nodules (0.4%) that may represent lymphoma/ malignancy and would need referral for definitive diagnosis and management.

In our study, half the images reported as abnormal by the field clinical officers were subsequently reported by our radiologists as normal. This finding is not surprising as TB prevalence surveys by design encourage high sensitivity with field officers having a low threshold for identification of abnormalities and referral for sputum-based testing to maximise sensitivity in the initial prevalence survey screening stage.^9^ CAD software has been shown to have a role in TB screening activities especially prevalence surveys with diagnostic accuracy higher than clinical officers and comparable with expert radiologists.^18 31^ Inter-reader variability of CXR reporting is an acknowledged limitation and was also the case in our study with inter reader agreement moderate at 0.41. This moderate agreement can be partly explained by the limited clinical information available to our reporting radiologists. Though CAD for TB software has an accuracy equal to expert readers, the software provides TB risk scores only. To characterise non-TB findings, once TB has been excluded, a human reader remains the only current solution available. CAD for cardiomegaly^25^ is under development and may provide an automated solution for the detection of cardiomegaly in the future.

Our study used population-based national prevalence data and an explicit sampling approach to select images for review. Each image was read by two expert radiologists. Moderate inter-reader variability was mitigated by applying a third reader to resolve discrepancies. However, low specificity is an acknowledged issue with radiological classification. This was a retrospective study and we had limited clinical information available. HIV results were self-reported. Important information such as smoking history and pre-existing medical conditions were not collected during the survey. We were therefore not able to adequately correlate clinical symptoms or HIV serostatus with our findings. Although the prevalence survey protocol required those with other CXR abnormalities to be linked to a health facility within the cluster, we had no way of ascertaining if this was done, or obtaining data on final diagnosis and clinical outcome.

## Conclusion and recommendations

Our findings are strikingly similar to those of the 1940s study in Europe; that mass radiography can be used to tackle ‘fundamental problems of disease in the chest, both of the respiratory system and also of the heart’ and ‘aid in detection of early and treatable non-TB disease’.^5^ Currently, TB screening activities using CXR and CAD software are focused on finding abnormalities consistent with TB.^16^ As countries embark on TB ACF activities, they need to be aware that other respiratory and non-respiratory pathologies are likely to be as, or more prevalent, than active TB. Mass screening with CXR therefore offers opportunity to plan for and address multiple important diseases.^5 6^ Even though the algorithms or protocols, for example, in TB prevalence surveys do recommend that any other abnormalities should be referred as appropriate, there is no structured system for the detection and referral of such patients.^9 15^ Clear referral pathways, diagnostics and follow-up plans for non-TB pathology could be incorporated during the planning of TB prevalence surveys and ACF activities.^6^ Prospective data collection about non-TB conditions identified during TB screening, characterisation of these patients, exploration of individual and health systems implications of these diseases could assist with further planning. Our findings were consistent with complications of potential underlying NCDs, including chronic respiratory disease in the population. At primary care health facilities, prevention efforts for NCDs could be strengthened including health messaging.

## Data Availability

Data may be obtained from a third party and are not publicly available. The Kenya Division of National Tuberculosis, Leprosy and Lung Disease program is the custodian of the 2016 Kenya Tuberculosis Prevalence Survey data. Our study used the prevalence survey data set for secondary analysis.
