# Supplementary material for: ‘If not TB, what could it be?’ Chest X-ray findings from the 2016 Kenya Tuberculosis Prevalence Survey
Source: Thorax. 2021 Jan 27;76(6):607–14. doi: 10.1136/thoraxjnl-2020-216123 (PMC8223623; doi:10.1136/thoraxjnl-2020-216123)
Supplement: Supplementary data [file thoraxjnl-2020-216123supp001.pdf]

Supplementary material

Appendix 1: Study radiologists details

Radiologist	Qualifications	Experience in TB imaging	Country	Reported
1	Consultant radiologist)	16 years	Kenya	Pilot study, Main study
2	Consultant radiologist	19 years	Kenya	Pilot study, Main study
3	Consultant radiologist Fellowship in radio-oncology	15 years	Kenya	Pilot study, Main study
4	Consultant radiologist	10 years	Kenya	Main study
5	Consultant radiologist	10 years	Kenya	Main study
6	Chest, Head and Neck radiologist	3 years	United Kingdom	Main study
7	General Body Imaging Radiologist	1 year	United Kingdom	Main study
8	Consultant Radiologist: Chest and Oncological Imaging	30 years	United Kingdom	Main study
9	Specialist chest radiologist	20 years	United Kingdom	Pilot study, Main study
10	Thoracic radiologist	10 years	United Kingdom	Main study
11	Consultant radiologist	15 years	United Kingdom	Pilot study, Main study-Third reader
12	Thoracic radiologist	12 years	United Kingdom	Main study-Third reader

Appendix 2: List of selected differential diagnostic options for CXR reporting and definitions*

	Primary diagnosis	Definition
**Lung parenchyma**	**Active PTB**	Features of active PTB https://pubs.rsna.org/doi/full/10.1148/rg.275065176
	**Non-tuberculous Mycobacterial infection**	Features overlap with PTB. Consider NTM in smaller cavities + midzone predominance
	**Old / latent TB : < 1 lobe of damage / scarring**	Features of latent/healed PTB with total scarring / damage less than 1 lobe equivalent
	**Old / latent TB : 1-2 lobes of damage / scarring**	Features of latent/healed PTB with total scarring / damage equal to 1-2 lobe equivalent
	**Old / latent TB : destroyed lung**	Complete destruction of 1 whole lung equivalent due to PTB
	**Pneumonia, not typical for PTB**	Suspected pneumonia, but without typical TB or PCP features
	**Non-specific airspace opacification**	Any airspace opacification that cannot be classified under any of the above
	**Bronchiectasis: any type or distribution**	Localized or diffuse bronchial dilatation: tramline appearances +/- wall thickening
	**Broncho vascular inflammation: smoking / COPD type**	Coarse, thickened bronchovascular bundles in peri-hilar distribution.
	**Suspected Emphysema / Asthma**	Hyperexpansion +/- flattening of diaphragm +/- bullous changes. Select only if unequivocal.
	**Interstitial pattern / pulmonary oedema from cardiac failure**	Classic features of congestive cardiac failure with interstitial +/- pulmonary oedema
	**Interstitial pattern, other than oedema**	Any interstitial patterns, including miliary. No further specification required.

	**Chronic scarring/volume loss, likely not related to TB**	Chronic parenchymal banding/architectural distortion, pattern unlikely to be the result of PTB
	**Mass/nodules: probably malignant**	Malignant features: e.g. spiculation, lymphadenopathy, and/or lung metastases
	**Mass / nodules: indeterminate**	Indeterminate: no definite malignant or benign features
	**Mass / nodules: probably benign**	Benign features: calcified granulomas, hamartomas, association with old TB scarring.
	**Mycetoma**	Fungal ball in a pre-existing cavity
	**Suspected Kaposi sarcoma**	"Flame like" opacities in peri-bronchial distribution +/- large effusions
**Pleura**	**Pleural effusion/ thickening / calcification: insignificant**	Small and likely potentially clinically irrelevant.
	**Pleural effusion: significant**	Size that is potentially clinically relevant, including in cardiac failure
	**Pleural thickening / calcification: likely benign**	Post-infectious / hemothorax (commonly unilateral) or pleural plaques (bilateral)
	**Pleural thickening / calcification: potentially malignant**	More than 10 mm thickness, mediastinal pleura involved +/- associated pleural plaques.
**Mediastinum (excluding heart and great vessels)**	**Mass, indeterminate**	Any mediastinal mass, other than suspected lymphoma/EPTB or goitre
	**Suspected Lymphoma**	Large volume adenopathy (+/- lung pathology), including bilateral hilar and extensive
	**Suspected EPTB**	Unilateral hilar adenopathy and mediastinal adenopathy (+/- lung pathology)
	**Goitre**	Superior mediastinal mass, extending to the neck with tracheal deviation
	**Spinal/para-spinal pathology**	Spinal/paraspinal mass or destruction

	Primary diagnosis	Definition
**Heart & great vessels**	**Cardiomegaly**	Cardiothoracic ratio > 0.5
	**Cardiac pathology: Other**	Any classic features, other than enlargement.
	**Pericardial effusion**	Globular enlargement of cardiac shadow
	**Aorta atherosclerosis / elongation**	Subjective elongation/calcification of aorta
	**Pulmonary arterial hypertension (PAH)**	Elevated cardiac apex, enlarged right atrium and pulmonary arteries, pruning of peripheral vessels
	**Aortic/Pulmonary artery pathology: other**	Any other than atherosclerotic / PAH
**Chest wall**	**Free text**	Any abnormalities of bones and chest wall

***Adapted from Fleischner Society guidelines** [19]

Appendix 3:

PILOT STUDY

We conducted a pilot study from December 2018 to January 2019, to trial and refine a list of selected Chest X-ray diagnoses, to refine standard operating procedures for reporting and to estimate a sample size for the main study.

Pilot study methods

We developed an on-line, study specific CXR reporting tool which comprised four major diagnostic categories: lung parenchyma, heart and great vessels, and the pleura and mediastinum. Under each heading, a pick list of most common expected diagnoses was given, taking into consideration Kenyan disease epidemiology and prevalence survey cohort. During reporting of the CXRs, readers were required to select one or more primary diagnoses as participants could have more than one diagnosis. , followed by the option of selecting up to two differential diagnoses. Due to limited specificity of CXR in many disease presentations, allowing for alternative diagnostic options was designed to capture those cases where a single, confident primary diagnosis could be not made. This study was aimed at deriving the prevalence of a final diagnosis rather than exploring image characteristics for each disease, the proforma did not capture detailed descriptions of each film.

Five radiologists were selected for the pilot: three consultant radiologists (MV, BM, BMM) from Kenya who had been part of the National TB Prevalence Survey team and two (JC, EJ) from the United Kingdom. Prior to the pilot readings, a test set of 20 X-rays was read independently by all radiologists, followed by discussion in a consensus meeting to ensure uniformity in reporting and application of the tool. Subsequently, each pilot image was read by a single radiologist only.

Pilot study sampling

We were unable to identify recent and relevant estimates from the literature to inform sample size estimates for prevalence of pulmonary abnormalities from similar settings in the pilot study. We therefore pragmatically set out to read 500 images (150 abnormal suggestive of TB; and 350 abnormal other) during the pilot, after which the detected prevalence of a set of predetermined pathologies was used to calculate a representative sample size for the main study. Once sampled, the labels of “Abnormal, suggestive of TB” and “Abnormal other” were removed to reduce the risk of bias.

Pilot study results

A total of 484 (97%) images were reported, 16 images were not read within the set time frame. The reporting was as follows: 288 (60%) as abnormal, 178 (37%) as normal, 8 (2.3%) were not interpretable. The abnormalities included: Heart and/or great vessel abnormalities 174 (37%), lung parenchyma abnormalities116 (24%), pleural abnormalities 39 (8.2%) and mediastinal abnormalities 8 (1.7%). In the images in the "abnormal other" category n=344, cardiomegaly was most prevalent at 122 (36%) and great vessel abnormalities at 79 (23%). In the "abnormal suggestive of TB" category n=122, old or latent TB was the most prevalent finding at 17(13.9%) and active PTB at 16(13.1) %. Cardiomegaly in this category was at 9(7%).

Appendix 4: Cohen`s Kappa scores for the expert radiologists inter-reader variability

**Radiologists**	**A**	**B**	**C**	**D**	**E**	**F**	**G**	**H**	**I**	**J**
**A**	1.000	0.372	0.178	0.000	-0.421	0.623	0.438	0.292	-0.429	0.016
**B**	0.372	1.000	0.659	0.322	0.459	0.700	0.475	0.114	0.503	0.214
**C**	0.178	0.659	1.000	0.046	0.409	0.814	0.215	0.204	0.298	0.224
**D**	0.000	0.322	0.046	1.000	0.765	0.500	0.488	0.678	0.232	0.500
**E**	-0.421	0.459	0.409	0.765	1.000	0.698	0.576	0.487	0.422	0.688
**F**	0.623	0.700	0.814	0.500	0.698	1.000	0.036	-0.227	0.529	0.800
**G**	0.438	0.475	0.215	0.488	0.576	0.036	1.000	0.175	0.455	0.500
**H**	0.292	0.114	0.204	0.678	0.487	-0.227	0.175	1.000	0.427	0.157
**I**	-0.429	0.503	0.298	0.232	0.422	0.529	0.455	0.427	1.000	0.620
**J**	0.016	0.214	0.224	0.500	0.688	0.800	0.500	0.157	0.620	1.000
